# Adverse pregnancy outcomes and long-term risk of peripheral artery disease: A cohort study

**DOI:** 10.1371/journal.pmed.1004821

**Published:** 2026-07-21

**Authors:** Casey Crump, Jingkai Wei, Jan Sundquist, Kristina Sundquist

**Affiliations:** 1 Department of Family and Community Medicine, McGovern Medical School, The University of Texas Health Science Center, Houston, Texas, United States of America; 2 Department of Epidemiology, School of Public Health, The University of Texas Health Science Center, Houston, Texas, United States of America; 3 Center for Primary Health Care Research, Department of Clinical Sciences, Lund University, Malmö, Sweden; 4 University Clinic Primary Care, Skåne University Hospital, Malmö, Sweden; University of Manchester, UNITED KINGDOM OF GREAT BRITAIN AND NORTHERN IRELAND

## Abstract

**Background:**

Women with adverse pregnancy outcomes have higher subsequent cardiovascular risks, but their long-term risks of peripheral artery disease (PAD) and potential causality are unclear. A better understanding of such risks is needed to improve early risk stratification, clinical care, and long-term cardiovascular health. We sought to determine long-term risks of PAD associated with 5 major adverse pregnancy outcomes in a large population-based cohort, and to assess for familial confounding using co-sibling analyses.

**Methods and findings:**

A national cohort study was conducted of all 2,201,446 women with a singleton delivery in Sweden in 1973–2015, followed up for PAD identified from nationwide inpatient and outpatient diagnoses linked through 2018. Cox regression was used to compute hazard ratios (HRs) for PAD associated with preterm delivery, small for gestational age, preeclampsia, other hypertensive disorders, and gestational diabetes, adjusting for other maternal factors (age, year of delivery, parity, socioeconomic factors, body mass index, smoking, and prior history of hypertension, diabetes, or hyperlipidemia). Co-sibling analyses assessed for potential confounding by shared familial (genetic and/or environmental) factors. In 54 million person-years of follow-up, 13,211 (0.6%) women were diagnosed with PAD (median age, 62 years). All adverse pregnancy outcomes were associated with significantly increased long-term risk of PAD. At 30–46 years following delivery, adjusted HRs for PAD associated with specific adverse pregnancy outcomes were: gestational diabetes, 3.83 (95% CI [3.20,4.59]; *p* < 0.001); small for gestational age, 1.74 (95% CI [1.65,1.83]; *p* < 0.001); other hypertensive disorders, 1.61 (95% CI [1.21,2.15]; *p* = 0.001); preterm delivery, 1.58 (95% CI [1.48,1.70]; *p* < 0.001); and preeclampsia, 1.28 (95% CI [1.20,1.37]; *p* < 0.001). These findings were largely unexplained by shared familial factors. Women with multiple adverse pregnancy outcomes had further increases in risk. This study was limited to Sweden and will need replication in other populations, and was based on diagnostic codes which may involve some misclassification.

**Conclusions:**

In this large national cohort, all adverse pregnancy outcomes were associated with increased risk for PAD up to 46 years later. Women with adverse pregnancy outcomes need early preventive actions and long-term clinical follow-up to reduce their risk of PAD and other associated cardiovascular diseases.

## Author summary

**Why was this study done?:**

- Women with pregnancy complications have higher future risk of heart disease, but their risk of peripheral artery disease (PAD) is unclear.
- A better understanding of such risk is needed to improve early prevention and long-term cardiovascular health in women.

**What did the researchers do and find?:**

- Risk of PAD was examined in a national cohort of >2 million mothers with up to 46 years of follow-up after delivery.
- All major pregnancy complications were associated with increased long-term risk of PAD.
- At 30–46 years after delivery, risk was elevated 3.8-fold among women with gestational diabetes, 1.7-fold among those with small for gestational age, 1.6-fold among those with preterm delivery, 1.6-fold among those with other hypertensive disorders, and 1.3-fold among those with preeclampsia.
- These findings were largely unexplained by shared familial factors.

**What do these findings mean?:**

- Women with pregnancy complications may warrant early cardiovascular risk assessment and long-term clinical follow-up given their higher subsequent risk of PAD.
- This study was limited to Sweden and will need replication in other populations, and was based on diagnostic codes which may involve some misclassification.

## Abstract 3

In this cohort study, Casey Crump and colleagues investigate whether adverse pregnancy outcomes increases the risk of peripheral artery disease in the mothers later in life using a Swedish national cohort.

## Introduction

Peripheral artery disease (PAD) affects >230 million people worldwide and is a strong predictor of future stroke, ischemic heart disease, and premature mortality [1]. Despite its high prevalence and clinical importance, PAD is understudied compared with other cardiovascular diseases. Established risk factors for PAD include traditional cardiovascular risk factors such as diabetes, hypertension, hyperlipidemia, smoking, and physical inactivity [1]. Adverse pregnancy outcomes have been identified as important risk factors for other cardiovascular diseases [2–4] and mortality [5] in women, but are less studied in relation to PAD. During their reproductive years, up to 30% of all women experience a major adverse pregnancy outcome (preterm delivery, small for gestational age, preeclampsia, other hypertensive disorders of pregnancy, or gestational diabetes) [3–8]. A better understanding of the long-term risks of PAD in such women is needed to improve their early risk stratification, clinical care, and long-term cardiovascular health.

Some [9–11] but not all [12,13] studies have suggested that preeclampsia or other hypertensive disorders of pregnancy are associated with increased risks of PAD in mid-adulthood. Preterm delivery and gestational diabetes also have been associated with increased risk of hospitalization for PAD [11]. However, hospital diagnoses may capture only the most severe cases and thus may not fully reflect overall PAD risk. To our knowledge, no large population-based studies have examined all major adverse pregnancy outcomes in the same cohort to assess their relative impacts on long-term risk of PAD ascertained in outpatient and inpatient settings. Moreover, it is unclear whether previously reported associations are explained by adverse pregnancy outcomes unmasking preexistent risk or eliciting new risk of PAD. Family-based designs that control for unmeasured shared familial (genetic and/or environmental) factors are needed to further elucidate potential causality.

To address these knowledge gaps, we conducted a national cohort study of over 2 million women in Sweden. Our goals were to: (1) determine risks for PAD associated with 5 major adverse pregnancy outcomes (preterm delivery, small for gestational age, preeclampsia, other hypertensive disorders of pregnancy, and gestational diabetes) using prospectively ascertained data in a large population-based cohort; (2) assess changes in such risks across the life course with up to 46 years of follow-up after delivery; and (3) assess for potential confounding by unmeasured shared genetic and environmental factors in families using co-sibling analyses. We hypothesized that women with adverse pregnancy outcomes would have long-term increased risks of PAD, and that such risks would be only partially explained by shared familial factors.

## Methods

### Study population

The Swedish Medical Birth Register contains prenatal and birth information for nearly all deliveries in Sweden since 1973. Using this registry, we identified 2,201,638 women who had a singleton delivery during 1973–2015 [3–7]. To improve internal comparability, only singleton deliveries were included, given the higher prevalence of adverse pregnancy outcomes and their different underlying causes in multiple gestation pregnancies. We excluded 192 (<0.1%) women with a prior diagnosis of PAD (identified using *International Classification of Diseases* [*ICD*] codes, as described below), leaving 2,201,446 women for inclusion in the study.

This study was approved by the Regional Ethical Review Board in Lund, Sweden (No. 2008/471 and later amendments). Participant consent was not required because this study only used pseudonymized registry-based secondary data. Dr. K. Sundquist had full access to the database population that was used to create the study population. The individual data were pseudonymized and therefore do not allow for the identification of any individuals. Further information about the data sources and their availability can be requested from the Swedish National Board of Health and Welfare.

### Adverse pregnancy outcome ascertainment

Five major adverse pregnancy outcomes were identified from prenatal and birth records in the Swedish Medical Birth Register [3–7]. Preterm delivery (gestational age <37 completed weeks) was based on maternal report of last menstrual period in the 1970s and ultrasound estimation starting in the 1980s and onward (>70% of the cohort). Small for gestational age was defined by infant birth weight <10^th^ percentile for gestational age. Preeclampsia (with or without further complications such as eclampsia), other hypertensive disorders of pregnancy (i.e., gestational or chronic hypertension), and gestational diabetes were identified from diagnostic codes in the Swedish Medical Birth, Hospital, and Outpatient Registers (Table A in S1 Appendix). Gestational age and birth weight in the Medical Birth Register have been found to be highly reliable [14,15]. Preeclampsia and other adverse pregnancy outcomes also have high validity with positive predictive values near 90% [16].

### Peripheral artery disease ascertainment

The study cohort was followed up for the earliest diagnosis of PAD from first delivery through December 31, 2018 (maximum follow-up time after delivery, 46 years; median, 25). PAD was identified using *ICD* codes in the Swedish Hospital and Outpatient Registers and primary care records (*ICD*-8/9 codes 443.8–443.9 during 1969–1996, and *ICD*-10 codes I70.2 and I73.8-I73.9 during 1997–2018). The Swedish Hospital Register contains all primary and secondary hospital discharge diagnoses from six populous counties in southern Sweden starting in 1964 and with nationwide coverage starting in 1987 [17]. PAD diagnoses in this registry have been reported to have a positive predictive value of 98% [18]. In addition, the Swedish Outpatient Register contains all diagnoses from specialty clinics with ~87% nationwide coverage starting in 2001 [19]. Primary care diagnoses previously collected by our group [20] were available for 20% of the Swedish population starting in 1998, 45% starting in 2001, and >90% starting in 2008 and onward.

### Covariates

Other maternal characteristics that may be associated with adverse pregnancy outcomes and PAD were identified using the Swedish Medical Birth Register and national census and diagnosis data, which were linked using a pseudonymous serial number. Maternal age was adjusted for in all analyses as the Cox model time scale (as described below). Covariates included the following maternal factors, with all time-varying factors updated for each pregnancy: calendar year of delivery (continuous and categorical in 5-year groups), parity (1, 2, 3, 4, and ≥ 5), education level (≤9, 10–12, and >12 years), employment (yes/no) and income (quartiles) in the year prior to pregnancy, country of origin (Sweden/other), and other PAD risk factors [1] which included smoking (0, 1–9, and ≥10 cigarettes/day) and body mass index (BMI; continuous and categorical [<18.5, 18.5–24.9, 25.0–29.9, and ≥30.0 kg/m^2^]) ascertained early in prenatal care, and pre-pregnancy history of hypertension, diabetes, or hyperlipidemia identified from nationwide diagnoses (Table A in S1 Appendix).

Maternal smoking and BMI were ascertained starting in 1982 and were available for 67% and 56% of women, respectively. Data were >97% complete for all other variables. Missing data were multiply imputed with 20 imputations using all other covariates and PAD as predictors. As an alternative to multiple imputation, a sensitivity analysis was performed that was restricted to women with complete data (*N* = 1,209,163).

### Statistical analysis

Cox proportional hazards regression was used to compute hazard ratios (HRs) and 95% confidence intervals (CIs) for subsequent risk of PAD associated with specific adverse pregnancy outcomes. These associations were examined across the maximum possible follow-up (up to 46 years after delivery) and within narrower intervals of follow-up (<10, 10–19, 20–29, and 30–46 years) among women still living in Sweden without a prior diagnosis of PAD at the beginning of the respective interval. All adverse pregnancy outcomes were modeled as time-dependent variables based on “ever” experiencing the respective outcome. For example, if a woman’s first delivery had no adverse pregnancy outcomes and her second was preterm with preeclampsia, she entered the preterm and preeclampsia exposure categories at the date of her second delivery. If a woman’s first and second deliveries were both preterm, she entered the preterm delivery exposure category at the date of her first delivery [3–7].

Maternal age was used as the Cox model time axis with age at each delivery as “time zero” (i.e., each singleton delivery was included as a separate observation, with exposures updated for each delivery). Women were censored at death as identified in the Swedish Death Register (*n* = 74,761; 3%) or at emigration as determined by absence of a Swedish residential address in census data (*n* = 92,904; 4%). For each adverse pregnancy outcome, two adjusted Cox models were performed that (1) adjusted for maternal sociodemographic factors, parity, and traditional PAD risk factors [1], and (2) further adjusted for all other adverse pregnancy outcomes. The proportional hazards assumption was assessed by examining log-log survival plots, and no substantial departures were found. Incidence rate differences and 95% CIs, attributable fraction in the exposed (AF_e_), and population attributable fraction (PAF) were computed for each adverse pregnancy outcome.

Co-sibling analyses were performed to assess for potential confounding by unmeasured familial (genetic and/or environmental) factors that may be shared determinants of adverse pregnancy outcomes and PAD. Shared environmental factors in families may include lifestyle factors such as physical activity and diet, or ambient exposures such as passive smoking and air pollution. These analyses included all 1,198,476 (54%) women with at least one full sister who had a singleton delivery (regardless of adverse pregnancy outcome status), and the same analysis was performed as the main analyses except for stratifying on sets of sisters. In the stratified Cox models, each stratum included a unique set of sisters identified by their mother’s and father’s pseudonymous serial numbers, modeled with their own baseline hazard function that reflects their shared genetic and environmental factors. Thus, these analyses examined associations between adverse pregnancy outcomes and PAD within each family, controlling for shared familial exposures [3–7]. In addition, these analyses were further adjusted for the same covariates as in the main analyses.

Other secondary analyses were performed to examine: (1) risk of PAD associated with spontaneous or medically indicated preterm delivery, which was systematically recorded starting in 1990 (*N* = 2,657,953 births among 1,452,455 women; maximum 29 years of follow-up); and (2) associations between the total number of adverse pregnancy outcomes (0, 1, 2, and ≥3; i.e., different adverse pregnancy outcomes in the same or different pregnancies, or the same adverse pregnancy outcome in different pregnancies) and PAD risk, adjusting for parity and other covariates.

All statistical tests were two-sided and used a significance level of 0.05. All analyses were conducted using Stata version 16.1. All analyses were planned a priori. This study is reported as per the Reporting of Studies Conducted using Observational Routinely-Collected Data (RECORD) guideline (S1 Checklist).

## Results

A total of 667,774 (30.4%) women experienced at least 1 adverse pregnancy outcome. The most common adverse pregnancy outcomes were small for gestational age (affecting 14.4% of women across all deliveries) and preterm delivery (8.9%). Table 1 shows characteristics of women with specific adverse pregnancy outcomes. Women with preterm or small for gestational age delivery were more likely to be younger at first delivery, have low education level or income, and/or to smoke. Women with preeclampsia were more likely to have low education level or income and/or high BMI. Women with other hypertensive disorders or gestational diabetes were more likely to be older at first delivery and/or have higher income or BMI.

**Table 1:** Baseline maternal characteristics by ever occurrence of an adverse pregnancy outcome, Sweden, 1973–2015.

	All women	Preterm delivery	Small for gestational age	Preeclampsia	Other hypertension	Gestational diabetes
	*N* = 2,201,446 (100.0%)	*N* = 195,646 (8.9%)	*N* = 316,045 (14.4%)	*N* = 132,990 (6.0%)	*N* = 34,358 (1.6%)	*N* = 36,268 (1.6%)
	*n* (%)	*n* (%)	*n* (%)	*n* (%)	*n* (%)	*n* (%)
**Age at first delivery (yrs)**						
<20	119,381 (5.4)	16,354 (8.4)	20,057 (6.3)	6,769 (5.1)	1,420 (4.1)	1,860 (5.1)
20–24	603,911 (27.4)	58,465 (29.9)	91,164 (28.9)	36,954 (27.8)	7,998 (23.3)	8,799 (24.2)
25–29	792,559 (36.0)	64,932 (33.2)	109,546 (34.7)	46,903 (35.3)	11,698 (34.0)	11,847 (32.7)
30–34	484,993 (22.0)	38,615 (19.7)	67,128 (21.2)	28,619 (21.5)	8,565 (24.9)	8,597 (23.7)
35–39	166,908 (7.6)	14,151 (7.2)	23,524 (7.4)	11,077 (8.3)	3,688 (10.7)	4,022 (11.1)
≥40	33,690 (1.5)	3,128 (1.6)	4,626 (1.5)	2,668 (2.0)	989 (2.9)	1,143 (3.2)
Unknown	4 (<0.1)	1 (<0.1)	0 (0.0)	0 (0.0)	0 (0.0)	0 (0.0)
**Year of first delivery**						
1973–1979	528,540 (24.0)	42,792 (21.9)	92,349 (29.2)	51,157 (38.5)	1,826 (5.3)	6,022 (16.6)
1980–1989	446,550 (20.3)	46,566 (23.8)	66,057 (20.9)	27,514 (20.7)	6,972 (20.3)	5,079 (14.0)
1990–1999	447,100 (20.3)	42,656 (21.8)	58,628 (18.5)	19,821 (14.9)	8,708 (25.3)	7,256 (20.0)
2000–2009	462,291 (21.0)	40,909 (20.9)	57,411 (18.2)	20,342 (15.3)	9,650 (28.1)	10,365 (28.6)
2010–2015	316,965 (14.4)	22,723 (11.6)	41,600 (13.2)	14,156 (10.6)	7,202 (21.0)	7,546 (20.8)
**Education (yrs)**						
≤9	304,585 (13.8)	30,430 (15.5)	54,073 (17.1)	21,412 (16.1)	2,962 (8.6)	6,460 (17.8)
10–12	970,729 (44.1)	91,399 (46.7)	144,551 (45.7)	62,882 (47.3)	15,162 (44.1)	15,949 (44.0)
>12	918,192 (41.7)	73,309 (37.5)	116,080 (36.7)	48,320 (36.3)	16,203 (47.2)	13,780 (38.0)
Unknown	7,940 (0.4)	508 (0.3)	1,341 (0.4)	376 (0.3)	31 (0.1)	79 (0.2)
**Employed**	1,899,941 (86.3)	166,530 (85.1)	265,855 (84.1)	120,353 (90.5)	31,256 (91.0)	28,616 (78.9)
**Income (quartile)**						
1st (highest)	535,853 (24.3)	43,339 (22.2)	65,782 (20.8)	26,395 (19.8)	12,441 (36.2)	10,286 (28.4)
2nd	535,743 (24.3)	46,024 (23.5)	73,737 (23.3)	33,025 (24.8)	8,498 (24.7)	8,377 (23.1)
3rd	536,299 (24.4)	48,517 (24.8)	82,610 (26.1)	38,616 (29.0)	6,628 (19.3)	7,352 (20.3)
4th (lowest)	535,298 (24.3)	53,886 (27.5)	83,834 (26.5)	31,985 (24.1)	6,425 (18.7)	9,332 (25.7)
Unknown	58,253 (2.6)	3,880 (2.0)	10,082 (3.2)	2,969 (2.2)	366 (1.1)	921 (2.5)
**Swedish-born**	1,807,217 (82.1)	161,372 (82.5)	252,665 (80.0)	115,157 (86.6)	30,092 (87.6)	25,931 (71.5)
**Body mass index (kg/m**^**2**^)						
<18.5	52,978 (2.4)	5,755 (2.9)	10,861 (3.4)	1,514 (1.1)	477 (1.4)	543 (1.5)
18.5–24.9	847,353 (38.5)	69,286 (35.4)	113,112 (35.8)	32,487 (24.4)	13,121 (38.2)	11,013 (30.4)
25.0–29.9	245,205 (11.1)	20,966 (10.7)	27,321 (8.6)	15,176 (11.4)	7,008 (20.4)	6,560 (18.1)
≥30.0	92,919 (4.2)	9,262 (4.7)	10,566 (3.3)	9,005 (6.8)	4,477 (13.0)	5,118 (14.1)
Unknown	962,991 (43.7)	90,377 (46.2)	154,185 (48.8)	74,808 (56.3)	9,275 (27.0)	13,034 (36.0)
**Smoking (cigarettes/day)**						
0	1,257,998 (57.1)	106,421 (54.4)	150,800 (47.7)	60,683 (45.6)	26,710 (77.7)	23,428 (64.2)
1–9	154,500 (7.0)	16,161 (8.3)	29,062 (9.2)	5,969 (4.5)	2,389 (7.0)	2,616 (7.2)
≥10	69,358 (3.2)	8,503 (4.3)	15,518 (4.9)	2,574 (1.9)	996 (2.9)	1,245 (3.4)
Unknown	719,590 (32.7)	64,561 (33.0)	120,665 (38.2)	63,764 (48.0)	4,263 (12.4)	8,979 (25.2)
**Hypertension**	2,007 (0.1)	460 (0.2)	451 (0.1)	506 (0.4)	726 (2.1)	361 (1.0)
**Diabetes**	7,957 (0.4)	2,603 (1.3)	551 (0.2)	1,632 (1.2)	449 (1.3)	6,109 (16.8)
**Hyperlipidemia**	1,719 (0.1)	273 (0.1)	202 (0.1)	191 (0.1)	114 (0.3)	416 (1.1)

**Fig 1:**
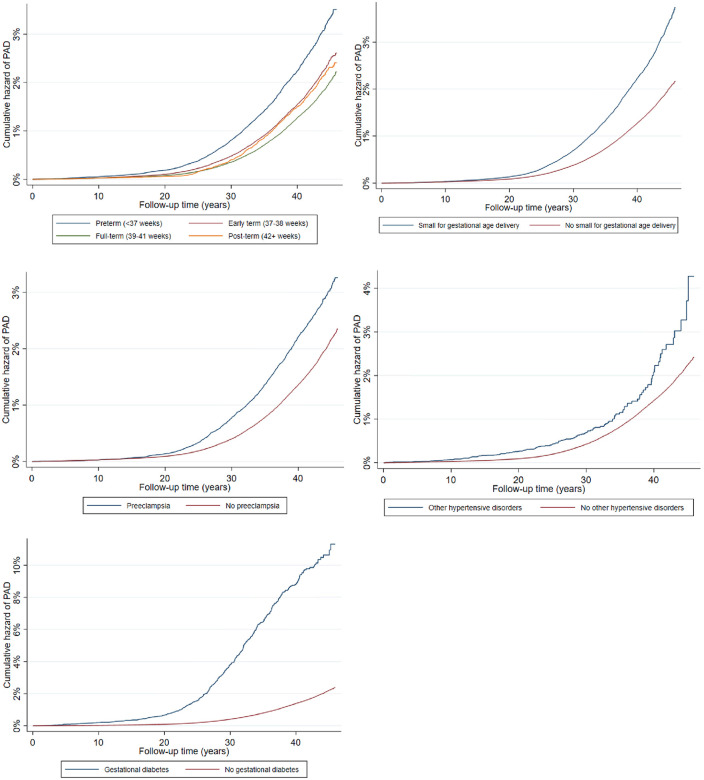
Cumulative hazard of peripheral artery disease (PAD) at 0–46 years after delivery associated with specific adverse pregnancy outcomes.

In 54 million person-years of follow-up, 13,211 (0.6%) women were diagnosed with PAD. A total of 3,604 (27.3%) diagnoses were made in inpatient settings, an additional 2,876 (21.8%) were made in specialty outpatient settings, and 6,731 (50.9%) were made only in primary care. 8,583 (65.0%) PAD diagnoses were identified by *ICD*-10 code I73.9, 4,042 (30.6%) by *ICD*-10 code I70.2, and 586 (4.4%) by other related codes (*ICD*-10 I70.3, *ICD*-8/9 443.8–443.9). The median age at first delivery was 27 (interquartile range [IQR], 23–30), at PAD diagnosis was 62 (IQR, 54–68), and at end of follow-up was 53 (IQR, 43–64) years. The median follow-up time in women who survived to the end of the follow-up period was 27 (IQR, 14–39) years. Absolute incidence rates for PAD by specific adverse pregnancy outcomes and follow-up time are reported in Table 2, and cumulative hazard curves are shown in Fig 1.

**Table 2:** Associations between adverse pregnancy outcomes and subsequent risk of peripheral artery disease (PAD).

	PAD cases	Rate^a^	Unadjusted model	Reduced model^b^	Full model^c^	Incidence rate difference^d^ (95% CI)	AF_e_^e^	PAF^f^
			HR (95% CI)	HR (95% CI)	HR (95% CI)			
**Up to 46 years after delivery**								
Preterm delivery	1,820	40	2.01 (1.91, 2.12)	1.83 (1.73, 1.93)	1.73 (1.64, 1.83)	18.5 (16.6, 20.4)	46.40%	9.20%
Small for gestational age	3,327	40.5	1.86 (1.78, 1.93)	1.76 (1.69, 1.83)	1.72 (1.65, 1.79)	19.0 (17.6, 20.5)	47.00%	11.80%
Preeclampsia	1,662	43.7	1.51 (1.43, 1.58)	1.48 (1.41, 1.56)	1.39 (1.32, 1.46)	20.8 (18.7, 23.0)	47.70%	6.00%
Other hypertensive disorders	153	25.7	1.72 (1.47, 2.02)	1.49 (1.26, 1.77)	1.31 (1.11, 1.55)	1.3 (−2.8, 5.4)	5.20%	0.10%
Gestational diabetes	519	118.7	3.24 (2.84, 3.68)	2.99 (2.62, 3.42)	2.87 (2.51, 3.29)	95.1 (84.9, 105.4)	80.10%	3.10%
**<10 years after delivery**								
Preterm delivery	82	5.4	2.18 (1.71, 2.77)	1.99 (1.53, 2.60)	2.02 (1.55, 2.64)	3.0 (1.8, 4.2)	55.10%	11.20%
Small for gestational age	100	3.4	1.35 (1.08, 1.67)	1.50 (1.19, 1.90)	1.53 (1.20, 1.94)	0.8 (0.1, 1.6)	24.80%	4.50%
Preeclampsia	33	2.6	0.97 (0.68, 1.37)	0.88 (0.59, 1.33)	0.74 (0.49, 1.12)	−0.1 (−1.0, 0.9)	(2.6%)^g^	(0.2%)^h^
Other hypertensive disorders	17	6.9	2.38 (1.47, 3.86)	1.56 (0.90, 2.70)	1.43 (0.82, 2.49)	4.3 (1.0, 7.6)	62.00%	1.90%
Gestational diabetes	37	20	2.24 (1.29, 3.86)	1.94 (1.07, 3.51)	1.84 (1.01, 3.35)	17.4 (11.0, 23.9)	87.40%	5.90%
**10**–**19 years after delivery**								
Preterm delivery	185	13.3	2.63 (2.22, 3.11)	1.96 (1.64, 2.34)	1.81 (1.52, 2.17)	8.1 (6.2, 10.1)	61.00%	16.80%
Small for gestational age	221	9.7	1.66 (1.43, 1.93)	1.65 (1.41, 1.92)	1.53 (1.31, 1.79)	3.7 (2.4, 5.1)	38.50%	8.60%
Preeclampsia	110	10.7	1.65 (1.36, 2.02)	1.80 (1.46, 2.21)	1.51 (1.23, 1.86)	4.5 (2.5, 6.5)	42.10%	4.70%
Other hypertensive disorders	36	18.8	2.81 (2.01, 3.91)	1.61 (1.13, 2.29)	1.55 (1.02, 2.05)	12.4 (6.3, 18.6)	66.20%	2.40%
Gestational diabetes	60	43.8	1.00 (0.70, 1.45)	0.80 (0.55, 1.17)	0.79 (0.54, 1.16)	37.6 (26.5, 48.7)	85.90%	5.20%
**20**–**29 years after delivery**								
Preterm delivery	591	58.6	2.40 (2.19, 2.64)	2.17 (1.98, 2.39)	2.00 (1.82, 2.21)	32.6 (27.7, 37.4)	55.60%	14.00%
Small for gestational age	911	53.2	2.00 (1.86, 2.16)	1.86 (1.72, 2.01)	1.81 (1.68, 1.96)	25.5 (21.9, 29.1)	47.90%	12.60%
Preeclampsia	502	61.7	1.95 (1.77, 2.14)	1.87 (1.69, 2.06)	1.67 (1.52, 1.84)	32.4 (26.9, 37.9)	52.50%	7.60%
Other hypertensive disorders	49	40.6	1.36 (1.02, 1.80)	1.24 (0.92, 1.68)	1.08 (0.80, 1.45)	9.0 (−2.4, 20.5)	22.20%	0.30%
Gestational diabetes	210	286.2	4.70 (3.80, 5.79)	4.35 (3.50, 5.40)	4.04 (3.26, 5.01)	256.2 (217.5, 294.9)	89.50%	5.40%
**30**–**46 years after delivery**								
Preterm delivery	962	150.6	1.71 (1.60, 1.84)	1.66 (1.54, 1.78)	1.58 (1.48, 1.70)	52.7 (42.8, 62.6)	35.00%	5.90%
Small for gestational age	2,095	163.6	1.83 (1.74, 1.92)	1.76 (1.68, 1.86)	1.74 (1.65, 1.83)	68.1 (60.7, 75.5)	41.60%	10.60%
Preeclampsia	1,017	145.8	1.36 (1.27, 1.45)	1.33 (1.25, 1.42)	1.28 (1.20, 1.37)	42.8 (33.5, 52.1)	29.40%	3.60%
Other hypertensive disorders	51	133.2	1.67 (1.27, 2.20)	1.83 (1.37, 2.44)	1.61 (1.21, 2.15)	26.4 (−10.2, 63.0)	19.80%	0.10%
Gestational diabetes	212	512.6	4.03 (3.37, 4.82)	3.91 (3.27, 4.68)	3.83 (3.20, 4.59)	407.9 (338.9, 477.0)	79.60%	2.10%

^a^PAD incidence rate per 100,000 person-years.

^b^Adjusted for maternal age, year of delivery, parity, education, employment, income, country of origin, BMI, smoking, and prior history of hypertension, diabetes, or hyperlipidemia.

^c^Adjusted for the same covariates as above and all other adverse pregnancy outcomes.

^d^Incidence rate difference per 100,000 person-years.

^e^Attributable fraction among the exposed.

^f^Population attributable fraction.

^g^Preventable fraction among the exposed.

^h^Population preventable fraction.

### Adverse pregnancy outcomes and risk of peripheral artery disease

Across the entire follow-up (up to 46 years after delivery), all adverse pregnancy outcomes were independently associated with increased risk of PAD. After adjusting for all other adverse pregnancy outcomes and covariates, the HRs for PAD associated with specific adverse pregnancy outcomes were: gestational diabetes, 2.87 (95% CI [2.51,3.29]; *p* < 0.001); preterm delivery, 1.73 (95% CI [1.64,1.83]; *p* < 0.001); small for gestational age, 1.72 (95% CI [1.65,1.79]; *p* < 0.001); preeclampsia, 1.39 (95% CI [1.32,1.46]; *p* < 0.001); and other hypertensive disorders, 1.31 (95% CI [1.11,1.55]; *p* = 0.001). The fully adjusted HRs (as above) were only slightly lower than those adjusted for maternal factors but not other adverse pregnancy outcomes (Table 2, Reduced model).

**Fig 2:**
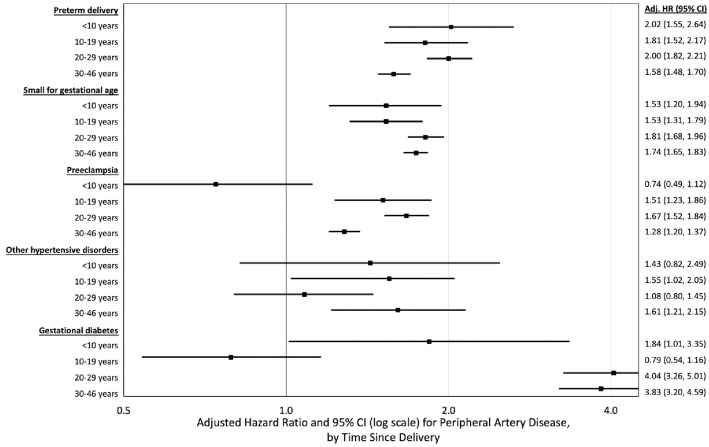
Adjusted hazard ratios (and 95% CIs) for associations between adverse pregnancy outcomes and peripheral artery disease by time since delivery, Sweden, 1973–2018.

Within 10 years following delivery, adjusted HRs for PAD were significantly elevated only among women with preterm delivery (2.02; 95% CI [1.55,2.64]; *p* < 0.001), gestational diabetes (1.84; 95% CI [1.01,3.35]; *p* = 0.04), or small for gestational age delivery (1.53; 95% CI [1.20,1.94]; *p* = 0.001). However, at 30–46 years after delivery, all 5 adverse pregnancy outcomes were associated with significantly elevated risk of PAD: gestational diabetes (adjusted HR, 3.83; 95% CI [3.20,4.59]; *p* < 0.001), small for gestational age (1.74; 95% CI [1.65,1.83]; *p* < 0.001), other hypertensive disorders (1.61; 95% CI [1.21,2.15]; *p* = 0.001), preterm delivery (1.58; 95% CI [1.48,1.70]; *p* < 0.001), and preeclampsia (1.28; 95% CI [1.20,1.37]; *p* < 0.001). Figure 2 shows adjusted HRs for PAD by time since delivery for specific adverse pregnancy outcomes.

All incidence rate differences (i.e., excess PAD risk) increased with longer follow-up to older ages (Table 2). Gestational diabetes was associated with the highest excess risk of PAD (e.g., 408 cases per 100,000 person-years at 30–46 years after delivery). Small for gestational age accounted for the largest percentage (12%) of PAD cases in this population of women across the entire follow-up, compared with 9% for preterm delivery, 6% for preeclampsia, and 3% for gestational diabetes (Table 2, PAFs).

### Co-sibling analyses

Co-sibling analyses to control for unmeasured shared familial (genetic and/or environmental) factors resulted in partial attenuation of most but not all risk estimates, with wider confidence intervals, and the association with other hypertensive disorders was no longer significant (Table 3). Across the entire follow-up (up to 46 years after delivery), the adjusted HR for PAD associated with preterm delivery was 1.73 (95% CI [1.64,1.83]) in the primary analysis (Table 2) versus 1.62 (95% CI [1.40,1.89]) in the co-sibling analysis (Table 3), with small for gestational age was 1.72 (95% CI [1.65,1.79]) versus 1.56 (95% CI [1.39,1.75]), with preeclampsia was 1.39 (95% CI [1.32,1.46]) versus 1.32 (95% CI [1.13,1.55]), with other hypertensive disorders was 1.31 (95% CI [1.11,1.55]) versus 1.06 (95% CI [0.68,1.65]), and with gestational diabetes was 2.87 (95% CI [2.51,3.29]) versus 2.97 (95% CI [1.93,4.57]).

**Table 3:** Co-sibling analyses of adverse pregnancy outcomes and subsequent risk of peripheral artery disease (PAD).

	PAD cases	Non-stratified^a^	Sibling-stratified^a^
		HR (95% CI)^b^	HR (95% CI)^b^
**Up to 46 years after delivery**			
Preterm delivery	965	1.70 (1.58, 1.83)	1.62 (1.40, 1.89)
Small for gestational age	1,715	1.70 (1.60, 1.80)	1.56 (1.39, 1.75)
Preeclampsia	807	1.41 (1.31, 1.52)	1.32 (1.13, 1.55)
Other hypertensive disorders	93	1.39 (1.11, 1.74)	1.06 (0.68, 1.65)
Gestational diabetes	268	2.62 (2.17, 3.16)	2.97 (1.93, 4.57)
**<10 years after delivery**			
Preterm delivery	40	1.98 (1.38, 2.83)	3.39 (0.45, 25.72)
Small for gestational age	49	1.56 (1.13, 2.15)	1.15 (0.29, 4.50)
Preeclampsia	18	0.78 (0.45, 1.37)	2.85 (0.15, 53.01)
Other hypertensive disorders	7	1.73 (0.86, 3.47)	2.12 (0.04, 107.01)
Gestational diabetes	24	1.44 (0.66, 3.16)	7.41 (0.06, 870.00)
**10**–**19 years after delivery**			
Preterm delivery	81	1.77 (1.40, 2.25)	1.36 (1.14, 1.97)
Small for gestational age	215	1.52 (1.24, 1.86)	3.87 (1.51, 9.94)
Preeclampsia	43	1.58 (1.19, 2.08)	2.47 (0.72, 8.52)
Other hypertensive disorders	19	1.45 (0.90, 2.34)	0.66 (0.11, 4.10)
Gestational diabetes	29	0.63 (0.37, 1.07)	1.10 (0.04, 29.32)
**20**–**29 years after delivery**			
Preterm delivery	275	1.92 (1.68, 2.19)	1.35 (0.95, 1.91)
Small for gestational age	395	1.66 (1.49, 1.85)	1.90 (1.43, 2.52)
Preeclampsia	208	1.71 (1.49, 1.96)	2.15 (1.46, 3.18)
Other hypertensive disorders	36	1.33 (0.91, 1.93)	0.86 (0.33, 2.19)
Gestational diabetes	98	3.77 (2.79, 5.11)	4.14 (1.23, 13.86)
**30**–**46 years after delivery**			
Preterm delivery	569	1.57 (1.42, 1.74)	1.62 (1.26, 2.08)
Small for gestational age	1,056	1.77 (1.64, 1.90)	1.50 (1.25, 1.79)
Preeclampsia	538	1.29 (1.17, 1.42)	1.16 (0.91, 1.49)
Other hypertensive disorders	31	1.48 (1.00, 2.21)	1.34 (0.45, 3.96)
Gestational diabetes	117	3.87 (3.00, 4.99)	6.33 (2.46, 16.27)

^a^Among women with at least one full sister with a singleton delivery (*N* = 1,198,476).

^b^Adjusted for shared familial (genetic and/or environmental) factors, and additionally for maternal age, year of delivery, parity, education, employment, income, country of origin, BMI, smoking, prior history of hypertension, diabetes, or hyperlipidemia, and all other adverse pregnancy outcomes.

### Secondary analyses

Medically indicated preterm delivery was associated with an increased relative rate of PAD (adjusted HR, 2.16; 95% CI [1.82,2.56]; *p* < 0.001), but spontaneous preterm delivery was not (1.23; 95% CI [0.99,1.53]; *p* = 0.06), compared with full-term delivery (Table B in S1 Appendix).

Women who experienced multiple adverse pregnancy outcomes had further increases in PAD risk (Table C in S1 Appendix). At 30–46 years after delivery, the adjusted HRs for PAD associated with 1, 2, or ≥3 adverse pregnancy outcomes were 1.40 (95% CI [1.34,1.47]), 1.83 (95% CI [1.69,1.98]), and 2.77 (95% CI [2.32,3.30]) (*P* < 0.001 for linear trend).

As an alternative approach for missing data, a complete case analysis yielded similar results as the main findings and the conclusions were unchanged, except for a weaker association with gestational diabetes that remained significantly elevated (Text A in S1 Appendix).

## Discussion

In this large national cohort, all major adverse pregnancy outcomes were associated with increased long-term risks of PAD. Within 10 years following delivery, the relative rate of PAD was significantly elevated only among women with preterm delivery (2.0-fold), gestational diabetes (1.8-fold), or small for gestational age (1.5-fold). However, at 30–46 years after delivery, relative rates of PAD were elevated 3.8-fold among women with gestational diabetes, 1.7-fold among those with small for gestational age, 1.6-fold among those with preterm delivery or other hypertensive disorders, and 1.3-fold among those with preeclampsia. Co-sibling analyses indicated that these findings were largely unexplained by genetic or environmental factors that may be shared determinants of adverse pregnancy outcomes and PAD within families.

This study is one of the largest to date to examine multiple adverse pregnancy outcomes in relation to long-term PAD risk. A previous meta-analysis reported that preeclampsia was associated with a nonsignificant nearly 2-fold risk of PAD in 3 smaller cohort studies (pooled relative risk, 1.87; 95% CI [0.94,3.73]) [21]. A US study of 1,697 women (mean age 60) found that a history of hypertension during pregnancy was associated with a 1.6-fold odds of PAD measured by ankle-brachial index (odds ratio, 1.61; 95% CI [1.04,2.49]) [10]. A Danish study of 1 million women found that women with gestational diabetes had more than a 2-fold risk of PAD during a median 16 years of follow-up (adjusted HR, 2.19; 95% CI [1.65,2.90]) [22]. A Swedish study of 2.1 million women reported that all major adverse pregnancy outcomes except small for gestational age were associated with increased future risk of PAD during a median 22 years of follow-up, with adjusted HRs ranging from 1.8 (preeclampsia) to 2.3 (preterm delivery) [11]. However, PAD was assessed using only inpatient and mortality data [11], and thus excluded less severe cases diagnosed in outpatient settings which comprise the majority of all PAD cases.

The present study extends prior evidence by examining 5 major adverse pregnancy outcomes in relation to PAD diagnoses prospectively ascertained from both outpatient and inpatient settings, thus enabling more robust risk estimates for a large national cohort. The findings show that all major adverse pregnancy outcomes are associated with increased risks of PAD later in life. Importantly, some of those risks did not emerge until several decades after delivery (e.g., in women with other hypertensive disorders). Because this was a relatively young cohort, the risks of PAD following adverse pregnancy outcomes may be even higher as women reach older ages when PAD is more likely to manifest. Gestational diabetes was associated with the highest relative and absolute long-term risks. Women who experienced multiple adverse pregnancy outcomes also had further increases in PAD risk.

These findings largely persisted in co-sibling analyses that controlled for shared familial (genetic and/or environmental) exposures. The underlying mechanisms linking adverse pregnancy outcomes with future development of PAD are not well established. However, long-term microvascular changes such as endothelial dysfunction, impaired microvascular reactivity, and reduced capillary density have been observed in women with preterm delivery [23], hypertensive disorders [24–26], or gestational diabetes [27,28], and may predispose to PAD. Adverse pregnancy outcomes are also associated with future development of type 2 diabetes [6,29,30] and chronic hypertension [31–33], which are strong risk factors for PAD [1]. Early interventions to improve cardiometabolic health soon after an adverse pregnancy outcome may be crucial for reducing subclinical disease and the long-term risk of PAD.

The present study’s findings have important clinical implications. All major adverse pregnancy outcomes should now be recognized as long-term risk factors for PAD. High-quality preconception and prenatal care are important priorities to reduce adverse pregnancy outcomes and their long-term health consequences. When adverse pregnancy outcomes occur, they provide a crucial opportunity to identify high-risk women long before the development of PAD or other cardiovascular sequelae, thus enabling earlier preventive actions [34,35]. Such actions should include reduction of other known risk factors, including diabetes, hypertension, hyperlipidemia, smoking, and physical inactivity [1]. Women with an adverse pregnancy outcome need lifelong clinical follow-up for continued prevention, timely detection, and treatment of PAD and other associated cardiovascular diseases [2–5]. Those with multiple adverse pregnancy outcomes should be recognized as an even higher-risk group needing aggressive risk reduction and more intensive monitoring. Because the risks of PAD continue to increase several decades after an adverse pregnancy outcome, pregnancy complications should be routinely tracked in electronic health records to facilitate risk assessment, preventive actions, and timely treatment throughout a woman’s life span.

A key strength of the present study is its large national cohort design with prospectively ascertained pregnancy outcomes and up to 46 years of follow-up for PAD. The availability of highly complete nationwide birth and medical registry data, including both inpatient and outpatient diagnoses, helped minimize potential selection and ascertainment biases. The large size of this cohort enabled high statistical power for simultaneous assessment of 5 major adverse pregnancy outcomes. The results were controlled for multiple other maternal factors, as well as unmeasured shared familial factors using co-sibling analyses.

This study also had certain limitations. First, detailed clinical records were unavailable to validate PAD diagnoses, although high positive predictive values have been reported in the Swedish registries [17,18]. Second, outpatient diagnoses were available starting in 2001, resulting in underreporting of PAD especially in earlier years. Consequently, the absolute risks of PAD associated with adverse pregnancy outcomes may be higher than those reported. Third, despite good validity of adverse pregnancy outcomes in the Swedish data [16], some underreporting may exist, which could influence results toward the null hypothesis. Screening practices and diagnostic coding also changed during the study period, although delivery year was adjusted for in all analyses. Fourth, certain covariates were available only during prenatal care (e.g., BMI and smoking) or were assessed in the year prior to each pregnancy (e.g., income) and not later in life. Medical risk factors (e.g., pre-pregnancy hypertension and hyperlipidemia) were ascertained from nationwide diagnoses and were likely underreported because of undiagnosed cases. Lastly, this study was limited to Sweden and will need replication in other populations, including assessment of potential racial/ethnic differences when feasible.

In this large national cohort, all major adverse pregnancy outcomes were associated with increased risks for PAD up to 46 years after delivery. Women with gestational diabetes had the highest long-term risks. Women who experience an adverse pregnancy outcome need early preventive actions and long-term clinical follow-up to reduce their risk for PAD and other associated cardiovascular diseases.

## Supporting information

S1 Appendix Appendix Text and Tables. **Text A**. Sensitivity analyses. **Table A.** *ICD* codes for adverse pregnancy outcomes, peripheral artery disease, and medical risk factors. **Table B.** Spontaneous or medically indicated preterm delivery (1990–2015) and subsequent risk of peripheral artery disease through 2018. **Table C.** Associations between number of adverse pregnancy outcomes and subsequent risk of peripheral artery disease.
(PDF)

S1 Checklist The RECORD statement. (PDF)

## Abbreviations

BMI: body mass index
CIs: confidence intervals
HRs: hazard ratios
ICD: International Classification of Diseases
IQR: interquartile range
PAD: peripheral artery disease
PAF: population attributable fraction
RECORD: Reporting of Studies Conducted using Observational Routinely-Collected Data.

## References

[1] Criqui MH, Matsushita K, Aboyans V, Hess CN, Hicks CW, Kwan TW. Lower extremity peripheral artery disease: contemporary epidemiology, management gaps, and future directions: a scientific statement from the American Heart Association. Circulation. 2021;144(9):e171–91. doi: 10.1161/CIR.0000000000001005 34315230 PMC9847212

[2] Lane-Cordova AD, Khan SS, Grobman WA, Greenland P, Shah SJ. Long-term cardiovascular risks associated with adverse pregnancy outcomes: JACC review topic of the week. J Am Coll Cardiol. 2019;73(16):2106–16. doi: 10.1016/j.jacc.2018.12.092 31023435

[3] Crump C, Sundquist J, McLaughlin MA, Dolan SM, Govindarajulu U, Sieh W, et al. Adverse pregnancy outcomes and long term risk of ischemic heart disease in mothers: national cohort and co-sibling study. BMJ. 2023;380:e072112. doi: 10.1136/bmj-2022-072112 36724989 PMC9890184

[4] Crump C, Sundquist J, Sundquist K. Adverse pregnancy outcomes and long-term risk of heart failure in women: national cohort and co-sibling study. JACC Heart Fail. 2025;13(4):589–98. doi: 10.1016/j.jchf.2024.11.004 39846910 PMC11981847

[5] Crump C, Sundquist J, Sundquist K. Adverse pregnancy outcomes and long-term mortality in women. JAMA Intern Med. 2024;184(6):631–40. doi: 10.1001/jamainternmed.2024.0276 38619848 PMC11019441

[6] Crump C, Sundquist J, Sundquist K. Long-term risk of type 2 diabetes after preterm delivery or hypertensive disorders of pregnancy. Obstet Gynecol. 2024;144(5):697–705. doi: 10.1097/AOG.0000000000005604 38723259 PMC11874906

[7] Crump C, Sundquist J, Sundquist K. Adverse pregnancy outcomes and long-term risk of chronic kidney disease in women: national cohort and co-sibling study. Am J Obstet Gynecol. 2024;230(5):563.e1-563.e20. doi: 10.1016/j.ajog.2023.10.008 37827269 PMC11006822

[8] Fraser A, Nelson SM, Macdonald-Wallis C, Cherry L, Butler E, Sattar N, et al. Associations of pregnancy complications with calculated cardiovascular disease risk and cardiovascular risk factors in middle age: the Avon Longitudinal Study of Parents and Children. Circulation. 2012;125(11):1367–80. doi: 10.1161/CIRCULATIONAHA.111.044784 22344039 PMC3323835

[9] Ray JG, Vermeulen MJ, Schull MJ, Redelmeier DA. Cardiovascular health after maternal placental syndromes (CHAMPS): population-based retrospective cohort study. Lancet. 2005;366(9499):1797–803. doi: 10.1016/S0140-6736(05)67726-4 16298217

[10] Weissgerber TL, Turner ST, Bailey KR, Mosley TH Jr, Kardia SLR, Wiste HJ, et al. Hypertension in pregnancy is a risk factor for peripheral arterial disease decades after pregnancy. Atherosclerosis. 2013;229(1):212–6. doi: 10.1016/j.atherosclerosis.2013.04.012 23659871 PMC3694211

[11] Täufer Cederlöf E, Lundgren M, Lindahl B, Christersson C. Pregnancy complications and risk of cardiovascular disease later in life: a nationwide cohort study. J Am Heart Assoc. 2022;11(2):e023079. doi: 10.1161/JAHA.121.023079 35014876 PMC9238523

[12] Hannaford P, Ferry S, Hirsch S. Cardiovascular sequelae of toxaemia of pregnancy. Heart. 1997;77(2):154–8. doi: 10.1136/hrt.77.2.154 9068399 PMC484665

[13] Wilson BJ, Watson MS, Prescott GJ, Sunderland S, Campbell DM, Hannaford P, et al. Hypertensive diseases of pregnancy and risk of hypertension and stroke in later life: results from cohort study. BMJ. 2003;326(7394):845. doi: 10.1136/bmj.326.7394.845 12702615 PMC153466

[14] Cnattingius S, Ericson A, Gunnarskog J, Källén B. A quality study of a medical birth registry. Scand J Soc Med. 1990;18(2):143–8. doi: 10.1177/140349489001800209 2367825

[15] Kallen B, Kallen K. The Swedish medical birth register—a summary of content and quality. Scand J Soc Med. 2003;18:143–8. doi: 10.1177/140349489001800209 2367825

[16] Cnattingius S, Källén K, Sandström A, Rydberg H, Månsson H, Stephansson O, et al. The Swedish medical birth register during five decades: documentation of the content and quality of the register. Eur J Epidemiol. 2023;38(1):109–20. doi: 10.1007/s10654-022-00947-5 36595114 PMC9867659

[17] Ludvigsson JF, Andersson E, Ekbom A, Feychting M, Kim J-L, Reuterwall C, et al. External review and validation of the Swedish national inpatient register. BMC Public Health. 2011;11:450. doi: 10.1186/1471-2458-11-450 21658213 PMC3142234

[18] Acosta S, Du Y, Borné Y, Gottsäter A. Differences in risk factor profiles for peripheral artery disease compared to coronary, cerebral and carotid artery. Sci Rep. 2025;15(1):3864. doi: 10.1038/s41598-025-88516-0 39890872 PMC11785722

[19] Swedish National Board of Health and Welfare. National Patient Register Stockholm, Sweden 2024 [accessed 08/26/2025]. Available from: https://www.socialstyrelsen.se/en/statistics-and-data/registers/national-patient-register/

[20] Sundquist J, Ohlsson H, Sundquist K, Kendler KS. Common adult psychiatric disorders in Swedish primary care where most mental health patients are treated. BMC Psychiatry. 2017;17(1):235. doi: 10.1186/s12888-017-1381-4 28666429 PMC5493066

[21] McDonald SD, Malinowski A, Zhou Q, Yusuf S, Devereaux PJ. Cardiovascular sequelae of preeclampsia/eclampsia: a systematic review and meta-analyses. Am Heart J. 2008;156(5):918–30. doi: 10.1016/j.ahj.2008.06.042 19061708

[22] Yu Y, Soohoo M, Sorensen HT, Li J, Arah OA. Gestational diabetes mellitus and the risks of overall and type-specific cardiovascular diseases: a population- and sibling-matched cohort study. Diabetes Care. 2022;45(1):151–9. doi: 10.2337/dc21-1018 34764208 PMC8753767

[23] Lane-Cordova AD, Gunderson EP, Carnethon MR, Catov JM, Reiner AP, Lewis CE, et al. Pre-pregnancy endothelial dysfunction and birth outcomes: the coronary Artery Risk Development in Young Adults (CARDIA) study. Hypertens Res. 2018;41(4):282–9. doi: 10.1038/s41440-018-0017-5 29449706 PMC6311125

[24] Hausvater A, Giannone T, Sandoval Y-HG, Doonan RJ, Antonopoulos CN, Matsoukis IL, et al. The association between preeclampsia and arterial stiffness. J Hypertens. 2012;30(1):17–33. doi: 10.1097/HJH.0b013e32834e4b0f 22134391

[25] Rangaswami J, Naranjo M, McCullough PA. Preeclampsia as a form of type 5 cardiorenal syndrome: an underrecognized entity in women’s cardiovascular health. Cardiorenal Med. 2018;8(2):160–72. doi: 10.1159/000487646 29627841 PMC5968275

[26] Strobl I, Windbichler G, Strasak A, Weiskopf-Schwendinger V, Schweigmann U, Ramoni A, et al. Left ventricular function many years after recovery from pre-eclampsia. BJOG. 2011;118(1):76–83. doi: 10.1111/j.1471-0528.2010.02780.x 21083867

[27] Knock GA, McCarthy AL, Lowy C, Poston L. Association of gestational diabetes with abnormal maternal vascular endothelial function. Br J Obstet Gynaecol. 1997;104(2):229–34. doi: 10.1111/j.1471-0528.1997.tb11051.x 9070145

[28] Lappas M. Markers of endothelial cell dysfunction are increased in human omental adipose tissue from women with pre-existing maternal obesity and gestational diabetes. Metabolism. 2014;63(6):860–73. doi: 10.1016/j.metabol.2014.03.007 24684825

[29] Li Z, Cheng Y, Wang D, Chen H, Chen H, Ming WK, et al. Incidence rate of type 2 diabetes mellitus after gestational diabetes mellitus: a systematic review and meta-analysis of 170,139 women. J Diabetes Res. 2020;2020:3076463. doi: 10.1155/2020/3076463 32405502 PMC7204113

[30] Zhu K, Wactawski-Wende J, Mendola P, Parikh NI, LaMonte MJ, Barnabei VM, et al. Adverse pregnancy outcomes and risk of type 2 diabetes in postmenopausal women. Am J Obstet Gynecol. 2024;230(1):93.e1-93.e19. doi: 10.1016/j.ajog.2023.07.030 37490991 PMC10803644

[31] Crump C, Sundquist J, Sundquist K. Preterm delivery and long-term risk of hypertension in women. JAMA Cardiol. 2022;7(1):65–74. doi: 10.1001/jamacardio.2021.4127 34643643 PMC8515256

[32] Xu J, Li T, Wang Y, Xue L, Miao Z, Long W, et al. The association between hypertensive disorders in pregnancy and the risk of developing chronic hypertension. Front Cardiovasc Med. 2022;9:897771. doi: 10.3389/fcvm.2022.897771 35872915 PMC9301072

[33] Behrens I, Basit S, Melbye M, Lykke JA, Wohlfahrt J, Bundgaard H, et al. Risk of post-pregnancy hypertension in women with a history of hypertensive disorders of pregnancy: nationwide cohort study. BMJ. 2017;358:j3078. doi: 10.1136/bmj.j3078 28701333 PMC5506851

[34] Sheiner E, Kapur A, Retnakaran R, Hadar E, Poon LC, McIntyre HD, et al. FIGO (International Federation of Gynecology and Obstetrics) postpregnancy initiative: long-term maternal implications of pregnancy complications-follow-up considerations. Int J Gynaecol Obstet. 2019;147 Suppl 1:1–31. doi: 10.1002/ijgo.12926 32323876

[35] Murray Horwitz ME, Fisher MA, Prifti CA, Rich-Edwards JW, Yarrington CD, White KO, et al. Primary care-based cardiovascular disease risk management after adverse pregnancy outcomes: a narrative review. J Gen Intern Med. 2022;37(4):912–21. doi: 10.1007/s11606-021-07149-x 34993867 PMC8734553

