# Supplementary material for: Adverse pregnancy outcomes and long-term risk of peripheral artery disease: A cohort study
Source: PLoS Med. 2026 Jul 21;23(7):e1004821. doi: 10.1371/journal.pmed.1004821 (PMC13387525; doi:10.1371/journal.pmed.1004821)
Supplement: S1 Appendix — Text A. Sensitivity analyses. Table A. ICD codes for adverse pregnancy outcomes, peripheral artery disease, and medical risk factors. Table B. Spontaneous or medically indicated preterm delivery (1990–2015) and subsequent risk of peripheral artery disease through 2018. Table C. Associations between number of adverse pregnancy outcomes and subsequent risk of peripheral artery disease. (PDF) [file pmed.1004821.s001.pdf]

S1 APPENDIX

Contents

Text A. Sensitivity analyses. Page 2

Table A. *ICD* codes for adverse pregnancy outcomes, peripheral artery disease, and medical risk factors. Page 3

Table B. Spontaneous or medically indicated preterm delivery (1990-2015) and subsequent risk of peripheral artery disease through 2018. Page 4

Table C. Associations between number of adverse pregnancy outcomes and subsequent risk of peripheral artery disease. Page 5

Text A. Sensitivity analyses.

When restricting to women with complete data as an alternative to multiple imputation, the adjusted HRs for PAD at 0-46 years after delivery associated with specific adverse pregnancy outcomes were: 1.58 (95% CI [1.43,1.75]) for preterm delivery, 1.45 (95% CI [1.33,1.57]) for small for gestational age delivery, 1.48 (95% CI [1.32,1.66]) for preeclampsia, 1.48 (95% CI [1.32,1.66]) for other hypertensive disorders, and 1.48 (95% CI [1.32,1.66]) for gestational diabetes. When coding missing data as a separate category, the corresponding HRs were 1.71 (95% CI [1.62,1.80]), 1.69 (95% CI [1.62,1.76]), 1.39 (95% CI [1.32,1.47]), and 1.36 (95% CI [1.15,1.61]), and 2.88 (95% CI [2.52,3.29]).

Table A. *ICD* codes for adverse pregnancy outcomes, peripheral artery disease, and medical risk factors.

	ICD-8	*ICD*-9	*ICD*-10
**Adverse pregnancy outcomes**			
Preterm delivery[a]	--	--	--
Small for gestational age[a]	--	--	--
Large for gestational age[a]			
Preeclampsia	637	624.4-624.7	O14-O15
Other hypertensive disorders	400-404	401-405, 642.0-642.3, 642.9	I10-I15, O10-O11, O13, O16
Gestational diabetes	--[b]	648.0, 648.8	O24
**Peripheral artery disease**	443.8-443.9	443.8-443.9	I70.2, I73.8-I73.9
**Medical risk factors**			
Hypertension	400-404	401-405	I10-I15
Diabetes	250	250	E10-E14
Hyperlipidemia	272	272	E78

[a]Preterm delivery (<37 completed weeks), small for gestational age (infant birth weight <10th percentile for gestational age), and large for gestational age (infant birth weight >90th percentile for gestational age) were identified based on reported gestational age at birth and birth weight in the Swedish Medical Birth Register.

[b]*ICD*-8 was used prior to 1987 and did not include a specific code for gestational diabetes. For deliveries before 1987, gestational diabetes was alternatively defined by a first diagnosis of diabetes (*ICD*-8 code 250) after 15 weeks of gestation, consistent with American Diabetes Association (ADA) criteria (ADA Professional Practice Committee for Diabetes; *Diabetes Care*. 2026;49(Suppl. 1):S27-S49). This definition excludes diabetes diagnosed in early pregnancy because they likely represent previously undiagnosed diabetes.

ICD = International Classification of Diseases

Table B. Spontaneous or medically indicated preterm delivery (1990-2015) and subsequent risk of peripheral artery disease through 2018.

	PAD cases	Rate[a]	HR (95% CI)[b]	*P*
**Preterm**				
Spontaneous	87	13.5	1.23 (0.99, 1.53)	0.06
Medically indicated	174	32.4	2.16 (1.82, 2.56)	<0.001
**Full-term**	1,503	8.9	Reference	

[a]Incidence rate per 100,000 person-years.
[b]Adjusted for maternal age, year of delivery, parity, education, employment, income, country of origin, BMI, smoking, prior history of hypertension, diabetes, or hyperlipidemia, and all other adverse pregnancy outcomes.
CI = confidence interval, HR = hazard ratio, PAD = peripheral artery disease

Table C. Associations between number of adverse pregnancy outcomes and subsequent risk of peripheral artery disease.

	PAD cases	Rate[a]	HR (95% CI)[b]	*P*
**Up to 46 years after first delivery**				
0 APOs	6,357	19.3	Reference	
1 APO	5,174	28.7	1.42 (1.37, 1.48)	<0.001
2 APOs	1,370	47.7	2.07 (1.95, 2.20)	<0.001
≥3 APOs	310	73.5	2.88 (2.54, 3.25)	<0.001
Per each additional APO			1.43 (1.40, 1.47)	<0.001[c]
**<10 years after first delivery**				
0 APOs	292	2.2	Reference	
1 APO	184	2.9	1.36 (1.11, 1.66)	0.002
2 APOs	53	5.8	1.98 (1.40, 2.81)	<0.001
≥3 APOs	17	12.3	2.53 (1.41, 4.54)	0.002
Per each additional APO			1.38 (1.21, 1.57)	<0.001[c]
**10-19 years after first delivery**				
0 APOs	482	5.3	Reference	
1 APO	346	6.6	1.23 (1.07, 1.42)	0.004
2 APOs	120	14.0	2.01 (1.62, 2.50)	<0.001
≥3 APOs	46	33.7	2.40 (1.69, 3.40)	<0.001
Per each additional APO			1.35 (1.24, 1.47)	<0.001[c]
**20-29 years after first delivery**				
0 APOs	1,514	23.7	Reference	
1 APO	1,391	36.5	1.59 (1.47, 1.71)	<0.001
2 APOs	456	71.0	2.80 (2.51, 3.12)	<0.001
≥3 APOs	108	116.0	3.55 (2.88, 4.38)	<0.001
Per each additional APO			1.61 (1.54, 1.68)	<0.001[c]
**30-46 years after first delivery**				
0 APOs	4,069	91.0	Reference	
1 APO	3,253	121.2	1.40 (1.34, 1.47)	<0.001
2 APOs	741	160.3	1.83 (1.69, 1.98)	<0.001
≥3 APOs	139	257.4	2.77 (2.32, 3.30)	<0.001
Per each additional APO			1.38 (1.34, 1.42)	<0.001[c]

[a]PAD incidence rate per 100,000 person-years.
[b]Adjusted for maternal age, year of delivery, parity, education, employment, income, country of origin, BMI, smoking, prior history of hypertension, diabetes, or hyperlipidemia, and all other adverse pregnancy outcomes.
[c]A significant linear relationship was found between number of APOs and PAD risk (*P*<0.001). A separate likelihood ratio test for departure from linear trend was non-significant (*P*>0.05).
APO = adverse pregnancy outcome, CI = confidence interval, HR = hazard ratio, PAD = peripheral artery disease
